# Translational insights into interleukin-driven macrophage polarization as diagnostic biomarkers for atopic dermatitis—a narrative review

**DOI:** 10.3389/fmolb.2026.1846718

**Published:** 2026-06-03

**Authors:** Hao Wu, Xueyan Zhang

**Affiliations:** 1 Dermatology Department, Heilongjiang Armed Police Corps Hospital, Harbin, Heilongjiang, China; 2 Continuing Education Department, The First Affiliated Hospital of Heilongjiang University of Chinese Medicine, Harbin, Heilongjiang, China

**Keywords:** atopic dermatitis, interleukin, macrophage polarization, NF- κB/STAT6/NLRP3 crosstalk, pruritus, skin barrier

## Abstract

Atopic dermatitis (AD) is a chronic inflammatory skin disease driven by immune dysregulation and skin barrier impairment. As a core regulatory axis, interleukin-mediated macrophage polarization modulates the inflammatory cascade and barrier repair via NF-κB/STAT6/NLRP3 pathway crosstalk. This narrative review systematically summarizes the bidirectional regulatory mechanism of the interleukin-macrophage polarization axis in AD, clarifies the vicious cycle linking immune imbalance, inflammatory amplification and barrier damage, and updates targeted interventions including biological agents and natural products. We further analyze the translational challenges of this axis and propose future directions for precise stratified therapy. This review innovatively integrates the immune-barrier crosstalk mechanism and translational insights, providing a theoretical basis for pathogenesis-oriented precise treatment of refractory AD.

## 1 Introduction

Atopic dermatitis (AD) is a common chronic inflammatory skin disease. It manifests core clinical features including severe pruritus, defective skin barrier function and recurrent inflammation. The global incidence of this disease exhibits a continuous upward trend, and children and adolescents are particularly susceptible to this condition (Woo and Kim, 2023; Kim and Leung, 2018; Bieber et al., 2017). Conventional clinical treatments for AD consist of topical glucocorticoids, Janus kinase (JAK) inhibitors, calcineurin inhibitors and systemic biological agents (Chovatiya and Paller, 2021; Eichenfield et al., 2014; Frazier and Bhardwaj, 2020). These regimens can relieve clinical symptoms to a certain extent but are accompanied by a number of unavoidable limitations. Long-term use of topical medications may induce adverse reactions such as skin atrophy and telangiectasis (Mooney et al., 2015). Most systemic biological agents target a single cytokine, which leads to obvious individual differences in response rates among patients with severe or complex phenotypes. In addition, most therapeutic strategies fail to fundamentally reverse the core pathological states of skin barrier damage and immune imbalance, and this situation results in the persistent and recurrent progression of the disease (Sung et al., 2025; Wang et al., 2020; Jang et al., 2023).

The occurrence and progression of AD follow a pathological cycle in which impaired skin barrier, immune dysregulation and chronic inflammation interact with each other. External allergens or irritants that penetrate the defective skin barrier can activate innate immune cells in the skin. Macrophages show obvious abnormalities in their polarization status in this process. Excessive accumulation of classically activated pro-inflammatory (M1) phenotypes and functional deficiency of alternatively activated anti-inflammatory (M2) phenotypes jointly promote the amplification of inflammatory cascades (Huang Y. et al., 2024; Han et al., 2013; Lee et al., 2019). Abnormally activated immune cells release a variety of interleukins in large quantities. These cytokines continuously activate inflammatory signaling pathways on the one hand, which aggravates symptoms such as skin redness, swelling and pruritus. On the other hand, they inhibit the differentiation of keratinocytes and the expression of barrier-related proteins, thus further damaging the integrity of the skin barrier (Soumelis et al., 2002; Kim et al., 2023; Aries et al., 2016). The impaired skin barrier in turn aggravates allergen penetration and immune cell activation. This eventually forms an intractable pathological cycle and drives the chronic progression of the disease (Aries et al., 2016; Dickel et al., 2010).

The interleukin family serves as a core signaling molecule that connects immune dysregulation and inflammatory injury. It also acts as a key upstream factor in regulating the polarization direction of macrophages (Guo et al., 2024; Sang et al., 2021). Within the pathological microenvironment of AD, interleukins act as upstream core regulators to form a precise crosstalk network with macrophage polarization. Pro-inflammatory interleukins (IL-1β, IL-4, IL-6, IL-17A) drive macrophages toward M1 or pathological M2 deviation via NF-κB and STAT6 pathways, while anti-inflammatory IL-10 and IL-22 restore polarization balance to repair the skin barrier. This bidirectional regulatory crosstalk directly determines the intensity of inflammation and barrier repair efficiency, and has been validated as a core therapeutic target in recent preclinical studies (Holgado et al., 2023; Suchitha et al., 2024; Liddiard et al., 2006; Kupschke and Schenk, 2025). Targeting interleukins to regulate the balance of macrophage polarization can achieve multiple therapeutic effects at the same time. These effects include inflammation suppression, skin barrier repair and pruritus alleviation. This kind of intervention can block the pathological closed loop at the upstream level and make up for the deficiencies of existing therapeutic methods. It has therefore become a core research direction for novel therapeutic strategies of AD (Jang et al., 2023; Huang Y. et al., 2024; Kim et al., 2023).

Most current studies on AD focus on the functional analysis of a single interleukin or macrophage phenotype. These studies lack systematic integration of the regulatory network through which the interleukin family modulates macrophage polarization (Garlanda et al., 2013; Gu et al., 2022). The characteristics and complementarity of relevant research worldwide have also not been fully summarized. Meanwhile, several practical challenges persist in the translation of basic research outcomes into clinical applications. These challenges include low efficiency of skin-targeted drug delivery and poor compatibility with the heterogeneity of disease phenotypes. Based on the integration of evidence from high-quality domestic and international literature, this article systematically illustrates the expression profiles of the interleukin family in AD and the molecular mechanisms by which interleukins regulate macrophage polarization. It summarizes global research progress and intervention strategies, analyzes the obstacles to clinical translation and puts forward directions for future research. This review aims to provide theoretical support and novel perspectives for the precise treatment of AD.

## 2 General overview of interleukins and inflammatory network regulation in atopic dermatitis

### 2.1 Biological characteristics of interleukins and their secretory regulation in atopic dermatitis

Interleukins are a group of cytokines that mediate signal communication between immune cells. They can be divided into multiple families based on their structural and functional properties. Members of different families show distinct expression patterns and regulatory effects in AD. The IL-1 family includes key molecules such as IL-1β, IL-18 and IL-33 (Garlanda et al., 2013). These factors are mainly secreted by activated macrophages and keratinocytes. Their expression is markedly increased in skin lesions of patients with moderate-to-severe AD and they act as critical initiators of inflammatory responses (Dickel et al., 2010; Holgado et al., 2023; Ramos et al., 2024). The IL-2 family is dominated by IL-4 and IL-13 which represent classic Th2-type cytokines (Zhang and Su, 2023). These cytokines contribute to the development of allergic inflammation through activation of the STAT6 pathway. They also serve as important mediators that trigger abnormal macrophage polarization (Han et al., 2013; Liddiard et al., 2006). The IL-6 family consists of IL-6 and IL-31 (Jones and Jenkins, 2018). IL-6 acts as a central hub connecting inflammation and immune dysregulation. IL-31 is directly related to the manifestation of chronic pruritus in AD (Sung et al., 2025; Huong Nguyen et al., 2022; Jeong et al., 2015). The IL-10 family is represented by IL-10 and IL-22 which possess dual functions of anti-inflammatory activity and tissue repair. The expression of these cytokines is inadequate in AD lesions so that they cannot efficiently counteract inflammatory reactions (Kim et al., 2023; Aries et al., 2016; Han and Hyun, 2023). IL-17A and IL-25 which belong to the IL-17 family can drive macrophages to adopt a unique mixed polarization phenotype (Borowczyk et al., 2021). These cytokines participate in the amplification of inflammation during acute flares of the disease (Lee et al., 2019; Senra et al., 2019).

The secretion and regulation of interleukins in AD are influenced by multiple factors. The release of alarmins caused by skin barrier damage, colonization of *Staphylococcus aureus* and exposure to environmental pollutants can all contribute to the excessive release of pro-inflammatory interleukins and inhibit the expression of anti-inflammatory interleukins. These effects are mediated by the activation of several signaling pathways including NF-κB, MAPK and JAK/STAT (Aye et al., 2020; Liu et al., 2024; Fleitas et al., 2022). Multiple studies have confirmed that the ratio of pro-inflammatory interleukins to anti-inflammatory interleukins is significantly imbalanced in both skin lesions and peripheral blood of patients with AD. This imbalanced state acts as a vital molecular foundation for the persistent progression of the disease (Jang et al., 2023; Koppes et al., 2016).

### 2.2 Core features of inflammatory injury in atopic dermatitis and the pivotal role of macrophage polarization

Inflammatory injury in AD is characterized by chronic cutaneous inflammation, impaired skin barrier function and intractable pruritus. Massive inflammatory cell infiltration and sustained release of inflammatory factors can be observed in skin lesions. These changes lead to epidermal hyperplasia and dermal edema. The expression of barrier-related proteins including filaggrin and involucrin is significantly reduced. The rate of transepidermal water loss is also greatly increased (Sung et al., 2025; Kim et al., 2023; Gu et al., 2022). Chronic pruritus represents a core symptom that severely affects the quality of life of patients. This symptom is closely related to abnormal neuroimmune interactions mediated by interleukins such as IL-31, IL-4 and IL-13 (Kim et al., 2023; Jeong et al., 2015).

As central cellular components of cutaneous innate immunity, macrophages play a decisive role in the regulation of inflammation in AD (Hashimoto et al., 2023). The polarization status of these cells directly determines the developmental direction of inflammatory responses. Classically activated macrophages exhibit a pro-inflammatory phenotype. They mainly secrete pro-inflammatory mediators including IL-1β, IL-6 and TNF-α (Wang and Ma, 2019). These factors activate downstream inflammatory signaling pathways and further aggravate skin damage (Huang Y. et al., 2024; Ramos et al., 2024). Alternatively activated macrophages are supposed to exert anti-inflammatory effects and participate in tissue repair. However, these cells undergo pathological deviation in the microenvironment of AD. They lose their normal anti-inflammatory capacity and instead contribute to the persistence of inflammation and the transmission of pruritus (Han et al., 2013; Mommert et al., 2021). The disruption of macrophage polarization balance represents a key cellular mechanism underlying the refractory inflammation and impaired barrier repair in AD (Lee et al., 2019; Novak et al., 2013).

### 2.3 Dual roles of interleukins in the regulation of inflammatory networks in atopic dermatitis

Interleukins exert dual regulatory functions in the inflammatory network of AD (Table 1). Imbalance in these functions serves as a core event in the development of the disease. Pro-inflammatory interleukins mainly include IL-1β, IL-4, IL-6, IL-13, IL-17A, IL-25 and IL-33 (Choi et al., 2017; Son et al., 2023; Nabe, 2014). These factors promote abnormal phenotypic polarization of macrophages by activating pro-inflammatory signaling pathways within these cells. IL-1β and IL-6 activate the NF-κB pathway to drive M1 macrophage polarization and upregulate the expression of pro-inflammatory mediators such as iNOS and TNF-α; IL-4 and IL-13 trigger the JAK-STAT6 cascade to induce pathological M2 macrophage differentiation and upregulate CD206 and CCL18; meanwhile, IL-1β activates the NLRP3 inflammasome to promote caspase-1-mediated maturation and secretion of IL-1β, forming a pro-inflammatory amplification loop (Huang Y. et al., 2024; Liddiard et al., 2006; Garlanda et al., 2013; Choi et al., 2019). They also recruit inflammatory cells including neutrophils and eosinophils to infiltrate the skin lesions. These processes amplify inflammatory responses and damage the skin barrier (Lee et al., 2019; Soumelis et al., 2002; Senra et al., 2019). Anti-inflammatory interleukins mainly include IL-10, IL-19, IL-22 and IL-24 (Fujimoto and Azuma, 2019; D'Avino et al., 2026; Lee et al., 2024). These factors can inhibit excessive activation of macrophages and promote their differentiation into protective phenotypes. IL-10 exerts anti-inflammatory effects by directly blocking NF-κB signaling, thereby inhibiting M1 macrophage polarization and restoring the balance of macrophage subsets (Huang Y. et al., 2024). They also upregulate the expression of barrier-related proteins and accelerate the repair of skin tissue (Kim et al., 2023; Aries et al., 2016; Han and Hyun, 2023).

**TABLE 1:** Summary of the dual roles of interleukins in the inflammatory network of atopic dermatitis.

Interleukin	Family	Model	Pro-inflammatory/ Anti-inflammatory	Signaling pathway	Key molecular targets	Result	References
IL-1β	IL-1 family	THP-1 macrophages (*in vitro*); DNFB-induced AD mice (*in vivo*)	Pro-inflammatory	NLRP3/NF-κB	iNOS, TNF-α, IL-6	Dictamnine inhibits IL-1β-mediated M1 macrophage polarization and alleviates AD-like skin lesions	Huang Y. et al. (2024)
IL-18	IL-1 family	IL-18BP knockout mice (*in vivo*); AD mouse model (*in vivo*)	Pro-inflammatory	STAT1/NF-κB	IFN-γ, iNOS	Recombinant IL-18BP inactivates IL-18 and attenuates AD inflammation and macrophage activation syndrome	Jang et al. (2023)
IL-33	IL-1 family	Bone marrow-derived macrophages (*in vitro*); myeloid A20 knockout mice (*in vivo*)	Dual (Pro-inflammatory/Repair)	STAT1/NF-κB	A20, IFN-γ	A20 acts as a negative regulator of IL-33-STAT1 signaling and controls macrophage polarization direction	Holgado et al. (2023)
IL-4	IL-2 family	RAW264.7 macrophages (*in vitro*); AD mice (*in vivo*)	Pro-inflammatory (Pathological M2)	STAT6	TARC, CCL18, CD206	IL-4 drives macrophages into pathological M2 phenotype and exacerbates AD inflammation and pruritus	Han et al. (2013), Liddiard et al. (2006), Mommert et al. (2021)
IL-13	IL-2 family	HaCaT cells (*in vitro*); OVA-induced AD mice (*in vivo*)	Pro-inflammatory (Pathological M2)	JAK2/STAT6	MDC, Filaggrin	IL-13 inhibits filaggrin expression, impairs skin barrier and induces abnormal macrophage polarization	Kim et al. (2023), Yang Y. G. et al. (2025)
IL-5	IL-2 family	HL-60 cells (*in vitro*); BN rat airway inflammation model (*in vivo*)	Pro-inflammatory	JAK2	Eosinophil activation	YM-90709 blocks IL-5 receptor and suppresses eosinophil-mediated inflammatory response	Morokata et al. (2002), Morokata et al. (2004)
IL-6	IL-6 family	HaCaT cells (*in vitro*); AD mice (*in vivo*)	Pro-inflammatory	JAK/STAT3, NF-κB/MAPK	TSLP, TARC, MCP-1	IL-6 sustains M1 macrophage polarization and forms an inflammatory amplification loop in AD	Sung et al. (2025), Huong Nguyenet al. (2022), Cao et al. (2026)
IL-31	IL-6 family	AD mice (*in vivo*); HaCaT cells (*in vitro*)	Pro-inflammatory	JAK/STAT	Itch-related pathway, IL-6	IL-31 mediates chronic pruritus in AD and amplifies the pro-inflammatory function of macrophages	Jeong et al. (2015)
IL-17A	IL-17 family	Mouse skin macrophages (*in vitro*); AD mice (*in vivo*)	Pro-inflammatory (Mixed polarization)	NF-κB/p38	iNOS, Arg-1, IL-6	IL-17A induces M1/M2 mixed phenotype of macrophages and aggravates acute AD inflammation	Lee et al. (2019)
IL-25 (IL-17E)	IL-17 family	Human macrophages (*in vitro*); mouse skin inflammation model (*in vivo*)	Pro-inflammatory	p38/MAPK	Neutrophil infiltration, IL-1β	IL-25 activates macrophages and recruits neutrophils to exacerbate skin inflammation	Senra et al. (2019)
IL-10	IL-10 family	RAW264.7 macrophages (*in vitro*); AD mice (*in vivo*)	Anti-inflammatory	NF-κB	IL-1Ra, Arg-1	IL-10 inhibits M1 macrophage polarization and restores anti-inflammatory and tissue repair functions	Aries et al. (2016), Min et al. (2021)
IL-22	IL-10 family	HaCaT cells (*in vitro*); AD mice (*in vivo*)	Anti-inflammatory	JAK/STAT	Filaggrin, Involucrin	IL-22 promotes skin barrier repair and enhances the anti-inflammatory ability of macrophages	Han and Hyun (2023), Gao et al. (2018)
IL-26	IL-10 family (IL-20 subgroup)	Immune cells (*in vitro*)	Pro-inflammatory	JAK1/STAT1/STAT3	Macrophage differentiation	IL-26 regulates macrophage differentiation and participates in AD inflammatory progression	Suchitha et al. (2024)
IL-8	Other	HaCaT cells (*in vitro*); AD mice (*in vivo*)	Pro-inflammatory	NF-κB/STAT1	M1 macrophage chemotaxis	IL-8 recruits M1 macrophages to infiltrate AD lesions and amplifies local inflammation	Dickel et al. (2010), Choi et al. (2020)
IL-12	Other	Macrophages (*in vitro*); allergic contact dermatitis mice (*in vivo*)	Pro-inflammatory	STAT4	IFN-γ	Xanthohumol inhibits IL-12 expression and alleviates chronic allergic contact dermatitis	Cho et al. (2010)

AD, atopic dermatitis; Arg-1, Arginase 1; MCP-1, monocyte chemotactic protein-1; STAT4, signal transducer and activator of transcription.

Thymic stromal lymphopoietin (TSLP), a key upstream alarmin secreted by damaged keratinocytes, acts as a critical bridge connecting skin barrier injury and immune dysregulation (Soumelis et al., 2002). TSLP activates dendritic cells to promote the secretion of IL-4 and IL-13, which further amplify STAT6 pathway activation and pathological M2 macrophage polarization, forming a molecular crosstalk network between epithelial damage, cytokine secretion and macrophage dysfunction (Han et al., 2013). Under normal physiological conditions, pro-inflammatory and anti-inflammatory interleukins maintain a dynamic equilibrium to preserve cutaneous immune homeostasis. In AD, pro-inflammatory interleukins are expressed at a markedly dominant level while the functions of anti-inflammatory interleukins are significantly suppressed. The imbalance between these dual effects directly leads to disturbed macrophage polarization and sustained amplification of inflammation. This series of pathological changes eventually gives rise to a wide range of clinical manifestations (Jang et al., 2023; Holgado et al., 2023; Liddiard et al., 2006).

## 3 Interaction mechanism between abnormal macrophage polarization and interleukin network in atopic dermatitis

### 3.1 Polarization characteristics and functional imbalance of macrophages in atopic dermatitis

Macrophages in skin lesions of AD exhibit obvious characteristics of polarization imbalance (Pareek et al., 2026). These characteristics show variable patterns at different stages of the disease. During the acute flare stage of the disease, pro-inflammatory macrophages are overwhelmingly dominant in lesion tissues. These cells highly express signature molecules such as iNOS and secrete large amounts of pro-inflammatory factors including IL-1β, IL-6 and IL-18 (Zhang et al., 2025). They rapidly initiate and amplify inflammatory responses by activating the NF-κB pathway and the NLRP3 inflammasome pathway. This process leads to acute clinical manifestations such as skin redness, swelling and exudation (Huang Y. et al., 2024; Lee et al., 2019; Ramos et al., 2024). Studies have demonstrated that the number of IL-1β-secreting pro-inflammatory macrophages in skin lesions is positively correlated with disease severity in patients with severe AD (Ramos et al., 2024).

In the chronic stage of the disease, macrophages gradually shift toward an alternatively activated phenotype but display a pathologically deviated profile. Although these macrophages highly express alternatively activated markers such as CD206, they fail to secrete anti-inflammatory mediators like IL-10 in a normal manner (Demetrius et al., 2025). They instead release chemokines including CCL18 to recruit Th2 cells into skin lesions and sustain the chronic inflammatory state (Mommert et al., 2021; Novak et al., 2013). Synergistic effects of histamine and IL-4 can further enhance the activation of this pathological phenotype and contribute to the persistence of pruritus and inflammation (Mommert et al., 2021).

Disruption of macrophage polarization balance leads to multiple pathological consequences (Wu et al., 2025). Persistently released pro-inflammatory factors cause continuous damage to skin tissue. Abnormally polarized macrophages lose their ability to participate in tissue repair, which makes it difficult to restore impaired skin barrier integrity (Hu et al., 2024). These cells also secrete pruritogenic factors to aggravate chronic pruritus. A triple pathological state is thus formed that involves inflammation, barrier damage and persistent pruritus (Han et al., 2013; Lee et al., 2019; Novak et al., 2013).

### 3.2 Bidirectional regulation of the interleukin network on macrophage polarization in atopic dermatitis

The interleukin network acts as a core upstream signal that controls the polarization direction of macrophages (Divakaruni et al., 2018; Zhu et al., 2015). Interleukins belonging to different families exert bidirectional regulatory effects on macrophage polarization through specific signaling pathways. Pro-inflammatory interleukins show strong inducing effects on abnormal macrophage polarization. IL-1β and IL-18 promote the differentiation of macrophages into pro-inflammatory phenotypes by activating the STAT1 pathway and the NF-κB/NLRP3 inflammasome axis; the NLRP3 inflammasome mediates the cleavage of pro-IL-1β into mature IL-1β via caspase-1, which further activates NF-κB signaling to enhance the secretion of TNF-α and IL-6, aggravating inflammatory injury (Garlanda et al., 2013; Kim K. C. et al., 2024; Shimada et al., 2004). They also suppress the secretion of anti-inflammatory factors to enhance inflammatory responses (Jang et al., 2023; Holgado et al., 2023; Ramos et al., 2024). IL-4 and IL-13 drive macrophages to transform into a pathologically alternatively activated phenotype via the STAT6 pathway. These cells lose their normal tissue-repairing functions and instead contribute to sustaining allergic inflammation (Han et al., 2013; Liddiard et al., 2006). IL-17A can induce macrophages to exhibit a mixed status with both pro-inflammatory and anti-inflammatory phenotypes (Uttarkar et al., 2019). This special phenotype is enriched in skin lesions during acute exacerbations of AD. It releases both pro-inflammatory factors and angiogenic factors to aggravate inflammation and tissue remodeling (Lee et al., 2019). IL-25 can activate the p38 pathway in macrophages to promote the secretion of pro-inflammatory factors. It also recruits neutrophil infiltration and amplifies inflammatory responses in the skin (Senra et al., 2019).

Anti-inflammatory interleukins exert regulatory effects that maintain balance and facilitate tissue repair. IL-10 serves as a central anti-inflammatory mediator that blocks the activation of the NF-κB pathway (Huang Z. et al., 2024). It inhibits the differentiation of macrophages into pro-inflammatory phenotypes and upregulates the expression of anti-inflammatory factors to restore the immunoregulatory function of macrophages (Aries et al., 2016; Min et al., 2021). IL-22 does not directly regulate macrophage polarization but can enhance the anti-inflammatory capacity of macrophages (Yu and Li, 2022). It also acts on keratinocytes to promote the expression of barrier-related proteins and achieve synergistic effects of anti-inflammation and tissue repair (Han and Hyun, 2023; Gao et al., 2018). Several intervention studies using natural products have confirmed that increasing the levels of anti-inflammatory interleukins can effectively reshape the balance of macrophage polarization and alleviate pathological conditions in AD (Kim et al., 2023; Gu et al., 2022; Han et al., 2018).

### 3.3 Vicious cycle between macrophage polarization and interleukins

In AD, aberrant macrophage polarization and dysregulated interleukin networks form a self-reinforcing inflammatory vicious cycle (Kasraie et al., 2010). Pro-inflammatory macrophages secrete abundant IL-1β, IL-6 and IL-18, which trigger keratinocytes to release TSLP; TSLP further promotes dendritic cells to secrete IL-4/IL-13, aggravating abnormal macrophage polarization (Han et al., 2013; Soumelis et al., 2002; Elentner et al., 2009). Chemokines secreted by pathologically alternatively activated macrophages recruit more immune cells to infiltrate skin lesions. This process further amplifies the release of pro-inflammatory interleukins and aggravates polarization imbalance (Liddiard et al., 2006; Mommert et al., 2021).

**FIGURE 1:**
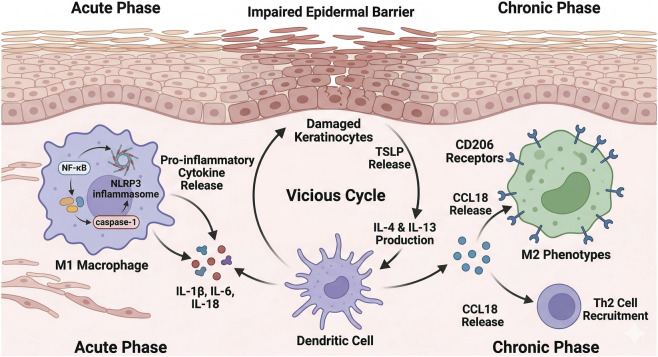
The interaction mechanism between macrophage polarization abnormalities and interleukin network in atopic dermatitis. The top epidermal barrier of cells (keratinocytes) is damaged. The visible break in the center illustrates the compromised skin barrier typical in Atopic Dermatitis (AD), which allows irritants in and moisture out. The specific skin cells surrounding the break that are releasing chemical “alarm” signals due to stress or damage. The Acute Phase (Left Side): M1 macrophages (purple cells) are immune cells with pro-inflammatory effects that reach the site of inflammation early. Intracellular Markers (NF-κB, NLRP3 inflammasome, caspase-1) are the internal signaling pathways and protein complexes within the M1 cell. They act as the machinery that detects danger and processes inflammatory signals into active cytokines. Specific pro-inflammatory cytokines IL-1 β, IL-6, and IL-18 (colored dots) are chemical messengers released by M1 macrophages to amplify acute inflammatory responses and activate other immune cells. The Bridge/Vicious Cycle (Center): Release of thymic stromal lymphopoietin (TSLP). This triggers an allergic/chronic immune cascade reaction. Dendritic cells (Spiky Purple Cells) are activated, which can effectively bridge the acute and chronic stages. The levels of IL-4 and IL-13 increase, forcing the immune environment to shift from acute reactions to chronic allergic reactions. The Chronic Phase (Right Side): This phase represents the long-term, non-resolving allergic state and tissue remodeling. M2 phenotype macrophages (green cells) are “distorted” by IL-4 and IL-13 to promote allergic reactions, itching, and tissue changes, rather than clearing the initial threat. CD206 receptor (blue Y-shaped) is a specific protein receptor found on the surface of M2 macrophages, which can serve as the main recognition marker for this particular cell type. M2 macrophages release (Teal Dots) a specific chemokine (chemoattractant) called CCL18. CCL18 attracts T helper 2 cells (small purple cells) to the skin. Once there, they produce more IL-4 and IL-13, locking the skin in a vicious cycle of chronic inflammation and preventing barrier healing.

Meanwhile, inflammatory responses mediated by interleukins continuously damage the skin barrier. Defects in the barrier further enhance stimulation from allergens and microorganisms. This process leads to further activation of macrophages and increased secretion of pro-inflammatory interleukins (Dickel et al., 2010; Hovnanian, 2013). The mutual reinforcement between cytokines and cellular phenotypes maintains persistent inflammatory status in AD (David Boothe et al., 2017). Disease recurrence frequently occurs even after conventional treatment (Sung et al., 2025; Wang et al., 2020; Jang et al., 2023). This vicious circle thus represents a core mechanism underlying the chronic progression of the disease (Figure 1).

## 4 Core mechanisms of treating atopic dermatitis via interleukin-mediated regulation of macrophage polarization

### 4.1 Targeting pro-inflammatory interleukins to block abnormal polarization and pathological progression

**FIGURE 2:**
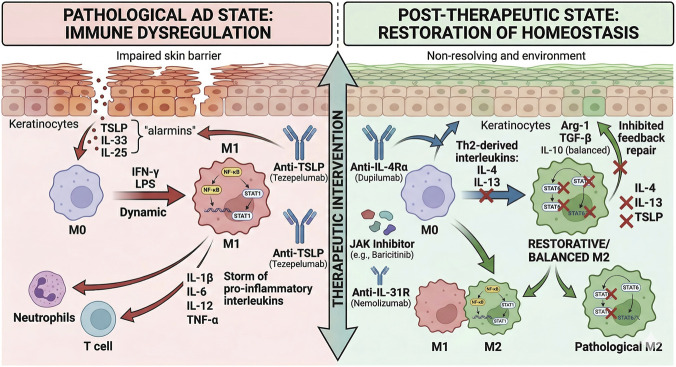
The core mechanism of leukocyte mediated macrophage polarization regulation in the treatment of atopic dermatitis. This illustration provides a clear, detailed overview of how targeted therapies can shift the AD immune environment from a dysregulated, pro-inflammatory state to a balanced, homeostatic, and self-repairing one. Left Side (Pathological State: Immune Dysregulation): This section summarizes the vicious cycle of AD, using inflamed reds, oranges, and purples. It visually captures the impaired skin barrier, keratinocytes releasing alarmins (TSLP, IL-33, IL-25), and the diverging paths of M0 macrophages towards pro-inflammatory M1 and pathological M2 skewing, complete with feedback loops that inhibit repair and promote tissue remodeling. Center Section (Therapeutic Intervention): A large central arrow separates the states, showcasing four specific therapeutic interventions with labeled icons and targets, as requested: Anti-TSLP (Tezepelumab): Targets initial alarmins; Anti-IL-31R (Nemolizumab): Targets pruning, breaking the itch-scratch-barrier breakdown loop; Anti-IL-4Rα (Dupilumab): Blocks pathological M2 skewing; JAK Inhibitor (e.g., Baricitinib): Provides broad, multi-pathway signaling blockade. Right Side (Post-Therapeutic State: Restoration of Homeostasis): This section utilizes cool greens and blues to visualize the healed microenvironment. A repaired, visibly intact skin barrier with proliferating keratinocytes. The number of M1/M2 cells decreased, and M0 cells successfully differentiated into “restorative/balanced M2” cells. An intracellular view showing the inhibition of pathological signaling pathways (e.g., STAT6, NF-κB, STAT1) by red ‘X' icons. IL-10 (balanced), Arg-1, TGF-β by restorative M2 cells to stimulate tissue repair.

We have summarized recent studies on the regulation of macrophages by different interleukins to improve AD inflammation, and the results are shown in Table 2. Targeting pro-inflammatory interleukins serves as a key strategy to reverse abnormal macrophage polarization and halt the pathological progression of AD (Figure 2). Such interventions block inflammation initiation and amplification upstream. IL-4/IL-13 signaling inhibition suppresses STAT6 activation, reduces pathological M2 macrophage polarization, and decreases TARC/MDC secretion (Shimada et al., 2004; Furue, 2020). These effects further decrease Th2 cell infiltration and pruritus transmission (Han et al., 2013; Liddiard et al., 2006; Mommert et al., 2021). Natural active ingredients including dictamnine and diosmetin can effectively ameliorate macrophage polarization imbalance by suppressing the expression of IL-4 and IL-13. They also alleviate AD-like symptoms in animal models (Huang Y. et al., 2024; Lee et al., 2020).

**TABLE 2:** Mechanisms of different interleukins regulating macrophages to ameliorate atopic dermatitis inflammation.

Interleukin	Family	Model	Signaling pathway	Key molecular targets	Main mechanism of action	Result	References
IL-6	IL-6 family	HaCaT cells (*in vitro*); Dermatophagoides farinae-induced AD mice (*in vivo*)	JAK1/STAT1/STAT3	TARC, MDC, Filaggrin	Inhibits IL-6/STAT3 signaling to regulate macrophage polarization and restore skin barrier	Ameliorates AD-like skin inflammation and reduces inflammatory cell infiltration	Sung et al. (2025)
IL-1β	IL-1 family	THP-1 macrophages (*in vitro*); DNFB-induced AD mice (*in vivo*)	NLRP3/NF-κB	iNOS, TNF-α, IL-6	Inhibits M1 macrophage polarization and promotes autophagy in inflammatory macrophages	Alleviates AD-like skin lesions and decreases pro-inflammatory cytokines release	Huang Y. et al. (2024)
IL-33	IL-1 family	Bone marrow-derived macrophages (*in vitro*); myeloid A20 knockout mice (*in* *vivo*)	STAT1/NF-κB	A20, IFN-γ	A20 acts as a negative regulator of IL-33-STAT1 signaling to control macrophage polarization direction	Determines IL-33-induced immune response and modulates AD-related inflammation	Holgado et al. (2023)
IL-6	IL-6 family	RAW264.7 macrophages (*in vitro*); DNFB-induced ACD mice (*in vivo*)	AKT/mTOR/MAPK	TNF-α, IL-1β	Suppresses LPS-induced macrophage inflammatory responses via enhancing autophagic flux	Ameliorates allergic contact dermatitis and AD-associated inflammation	Wang et al. (2020)
IL-4	IL-2 family	RAW264.7 macrophages (*in vitro*); DNCB-induced AD mice (*in vivo*)	JAK/STAT/MAPK	iNOS, TNF-α, IL-1β	Inhibits IL-4/LPS-induced macrophage activation and inflammatory cytokine secretion	Reduces AD skin lesion severity and macrophage infiltration	Lee et al. (2020)
IL-25 (IL-17E)	IL-17 family	Human macrophages (*in vitro*); mouse skin inflammation model (*in vivo*)	p38/MAPK	Neutrophil infiltration, IL-1β	Activates macrophages and recruits neutrophils via p38 signaling to enhance innate immunity	Exacerbates skin inflammation and promotes AD-related innate immune responses	Senra et al. (2019)
IL-4	IL-2 family	Mouse pulmonary macrophages (*in vitro*); TSLP-induced AD model (*in vivo*)	STAT6	CD206, Arg-1	Amplifies IL-13-dependent alternative (M2) macrophage polarization	Promotes allergic inflammation and participates in the pathogenesis of AD	Han et al. (2013)
IL-4	IL-2 family	Human monocyte-derived M2 macrophages (*in vitro*)	JAK/STAT6	CCL18, H2R	Histamine enhances IL-4-induced CCL18 expression in M2 macrophages via H2R	Aggravates AD inflammation by recruiting Th2 cells into skin lesions	Mommertet al. (2021)
IL-1β	IL-1 family	RAW264.7 macrophages (*in vitro*)	MAPK (p38/JNK/ERK)	IL-6, TNF-α	Suppresses MAPK activation to inhibit pro-inflammatory cytokine production in macrophages	Controls skin inflammation and relieves AD-related inflammatory damage	Kee et al. (2017)
IL-6	IL-6 family	RAW264.7 macrophages (*in vitro*)	NF-κB/MAPK	TNF-α, IL-6	Inhibits NF-κB and MAPK pathways to suppress macrophage inflammatory responses	Exerts anti-inflammatory activity in AD-related cutaneous inflammation	Ngo et al. (2020)
IL-17A	IL-17 family	Mouse skin macrophages (*in vitro*); AD mouse model (*in vivo*)	NF-κB/p38	iNOS, Arg-1, IL-6	Induces heterogeneous M1/M2 mixed phenotype polarization in macrophages	Promotes AD-like skin inflammation and tissue remodeling	Lee et al. (2019)
IL-18	IL-1 family	Keratinocytes (*in vitro*); AD *in vitro* models	NF-κB	TSLP, IL-8	Induces IL-10 secretion in macrophages to inhibit pro-inflammatory interleukins	Ameliorates AD inflammation and regulates local immune balance	Aries et al. (2016)
IL-6	IL-6 family	Activated macrophages (*in vitro*); oxazolone-induced dermatitis mice (*in vivo*)	NF-κB	TNF-α, IL-6	Inhibits pro-inflammatory cytokine production in activated macrophages	Protects mice from allergic contact dermatitis and AD-like inflammation	Min et al. (2023)
IL-4	IL-2 family	Murine macrophages (*in vitro*)	STAT6	TARC (CCL17)	Induces TARC expression via STAT6 binding to TARC gene promoter	Promotes macrophage-mediated chemotaxis and aggravates AD inflammation	Liddiard et al. (2006)
IL-5	IL-2 family	HL-60 cells (*in vitro*); human eosinophils (*in vitro*)	JAK2	Eosinophil activation	Blocks IL-5 binding to its receptor to inhibit eosinophil activation	Reduces eosinophil-mediated macrophage activation in AD	Morokata et al. (2002)
IL-18	IL-1 family	IL-18BP knockout mice (*in vivo*); AD mouse model (*in vivo*)	STAT1/NF-κB	IFN-γ, iNOS	Inactivates IL-18 to suppress M1 macrophage polarization and IFN-γ secretion	Attenuates AD inflammation and macrophage activation syndrome	Jang et al. (2023)
IL-1β	IL-1 family	Human AD skin tissues (*ex vivo*); macrophages (*in vitro*)	NLRP3/Caspase-1/GSDMD	ASC, CD68	Activates cutaneous inflammasome to promote IL-1β secretion in macrophages	Aggravates severe AD skin inflammation and tissue damage	Ramos et al. (2024)
IL-6	IL-6 family	RAW264.7 macrophages (*in vitro*)	ERK/p38 MAPK	TNF-α, IL-1β, iNOS	Inhibits MAPK phosphorylation to suppress pro-inflammatory cytokine secretion in macrophages	Relieves AD-related allergic inflammation and immune disorders	Min et al. (2021)
IL-5	IL-2 family	BN rats airway inflammation model (*in vivo*)	JAK2	Eosinophil infiltration	Inhibits antigen-induced eosinophil recruitment via IL-5 receptor blockade	Suppresses eosinophil-associated inflammation relevant to AD pathogenesis	Morokata et al. (2004)

AD, atopic dermatitis; Arg-1, Arginase 1; LPS, lipopolysaccharide; MCP-1, monocyte chemotactic protein-1; STAT4, signal transducer and activator of transcription.

Inhibition of IL-1β, IL-6 and IL-33 function can block the activation of pro-inflammatory macrophages and the NLRP3 inflammasome (Choi et al., 2019; Ge et al., 2025). It reduces the secretion of pro-inflammatory mediators including IL-1β, IL-6 and TNF-α and breaks the cycle of inflammatory amplification (Holgado et al., 2023; Ramos et al., 2024; Liu et al., 2024). Recombinant human IL-18 binding protein can directly inactivate IL-18 (Ihim et al., 2022). It markedly reduces the proportion of pro-inflammatory macrophages and alleviates symptoms of AD and macrophage activation syndrome (Jang et al., 2023). Extracts from Glycine soja and red ginseng can suppress the IL-6/STAT3 pathway. They reduce the infiltration of pro-inflammatory macrophages and simultaneously repair the skin barrier (Sung et al., 2025; Han et al., 2018).

Inhibition of IL-17A and IL-25 activity can reduce the generation of macrophages with a mixed polarization phenotype. It also decreases infiltration of neutrophils and eosinophils to alleviate acute inflammatory flares (Lee et al., 2019; Senra et al., 2019). Multiple studies have confirmed that such targeted interventions can simultaneously improve skin inflammation, pruritus and barrier damage. This approach therefore achieves multi-dimensional therapeutic effects (Huang Y. et al., 2024; Kim et al., 2023; Gu et al., 2022).

### 4.2 Enhancing anti-inflammatory interleukins to activate polarization balance and tissue repair

Enhancing the expression and function of anti-inflammatory interleukins can activate the protective polarization state of macrophages and simultaneously achieve the dual effects of anti-inflammation and skin barrier repair (Zhu et al., 2025). Upregulating the level of IL-10 can effectively inhibit the activation of pro-inflammatory macrophages and restore the anti-inflammatory functions of these cells. This intervention also reduces the release of inflammatory factors by keratinocytes (Aries et al., 2016; Min et al., 2021). Extracts of Aquaphilus dolomiae can induce macrophages to secrete IL-10, which significantly decreases the inflammatory level in AD models (Aries et al., 2016).

Upregulating IL-22 expression can enhance the anti-inflammatory capacity of macrophages and promote the expression of barrier-related molecules such as filaggrin and involucrin in keratinocytes, thus accelerating skin barrier repair (Han and Hyun, 2023; Gao et al., 2018). Natural products including lycium leaf extract and mulberry extract can reshape the balance of macrophage polarization by elevating IL-22 levels, thereby improving skin lesions and barrier function in mouse models of AD (Kim et al., 2023; Lee et al., 2018). Different from traditional anti-inflammatory treatments, such interventions not only suppress inflammation but also repair the skin barrier at its root, breaking the pathological cycle. This provides a novel strategy for the long-term remission of AD (Kim et al., 2023; Gu et al., 2022; Oh et al., 2021).

### 4.3 Downstream dual therapeutic effects

Intervention strategies that regulate macrophage polarization via interleukins can exert dual downstream therapeutic effects: anti-inflammation and tissue repair (Min et al., 2023). The anti-inflammatory effect is mainly reflected in inhibiting the activation of pro-inflammatory signaling pathways, reducing the release of inflammatory and chemotactic factors, decreasing inflammatory cell infiltration, and alleviating acute inflammation such as skin redness, swelling and exudation, as well as chronic pruritus (Wang et al., 2020; Huang Y. et al., 2024; Lee et al., 2020). Multiple animal experiments have confirmed that targeting the interleukin-macrophage axis can significantly reduce dermatitis scores and scratching frequencies in AD models (Sung et al., 2025; Kim et al., 2023; Oh et al., 2021).

The tissue repair effect is mainly reflected in restoring the normal differentiation of keratinocytes, upregulating the expression of barrier-related proteins such as filaggrin and loricrin, reducing transepidermal water loss, and repairing the damaged skin barrier (Gu et al., 2022). Interventions such as Glycine soja extract and total flavonoids from seabuckthorn can achieve both inflammation inhibition and barrier repair, verifying the clinical translational value of this dual effect (Sung et al., 2025; Gu et al., 2022). Such dual effects can ameliorate the core symptoms of AD at the pathological root and reduce the risk of disease recurrence (Jang et al., 2023; Kim et al., 2023; Oh et al., 2021).

## 5 Current research status worldwide

### 5.1 International research progress

International research focuses on the elucidation of molecular mechanisms underlying interleukin-mediated regulation of macrophage polarization and the clinical development of targeted biological agents (Kim R. W. et al., 2024). The core directions center on the development of single-target or multi-target interleukin inhibitors. Anti-IL-4Rα monoclonal antibodies can simultaneously block the signaling of IL-4 and IL-13 and inhibit pathological macrophage polarization. They have shown favorable efficacy in patients with moderate-to-severe AD and have become first-line biological agents in clinical practice. Anti-IL-13 monoclonal antibodies, anti-IL-33 monoclonal antibodies and other agents regulate macrophage polarization by specifically blocking single pro-inflammatory interleukins. These agents have significantly improved patients’ symptoms and quality of life in clinical trials (Eichenfield et al., 2014; Jang et al., 2023; Shimada et al., 2004), (Jang et al., 2023; Holgado et al., 2023; Morokata et al., 2002).

International research has also deeply explored the molecular regulatory networks of interleukin-mediated macrophage polarization, revealing the key roles of molecules such as A20 protein and CHI3L1, which provides a theoretical basis for the development of novel therapeutic targets (Holgado et al., 2023; Gu et al., 2025). Meanwhile, international research teams have focused on the phenotypic heterogeneity of AD. Studies on individualized targeted therapy have been conducted for patient groups with different interleukin expression profiles, aiming to improve treatment response rates (Koppes et al., 2016).

### 5.2 Domestic research progress

Domestic research is characterized by regulating the interleukin-macrophage polarization axis via natural products and traditional Chinese medicine (TCM), forming a complete research chain connecting basic research with the development of topical preparations. Studies on active ingredients of TCM play a dominant role. As the core component of Cortex Dictamni, dictamnine can inhibit M1-type macrophage polarization and promote autophagy, reduce the release of pro-inflammatory factors such as IL-1β and IL-6, and effectively alleviate AD-like skin lesions in animal models (Huang Y. et al., 2024). Flavonoids including Glycine soja stem and leaf extract, total flavonoids of seabuckthorn and quercetin glycosides can downregulate the expression of pro-inflammatory interleukins such as IL-6 and IL-1β by inhibiting the JAK/STAT or NF-κB signaling pathways, reshape macrophage polarization balance, and upregulate filaggrin expression to repair the skin barrier (Gu et al., 2022; Yang S. C. et al., 2025). Small-molecule natural products such as diosmetin and xanthone can directly suppress macrophage activation and inflammatory factor secretion, thus relieving skin inflammation and pruritus in animal models (Aye et al., 2020; Lee et al., 2020).

However, domestic TCM and natural product research still has prominent defects and obvious international research gaps. Most studies stay at the preclinical stage of cells and animals, with unclear active components, ambiguous core molecular targets, and lack of standardized quality control systems (Huang Y. et al., 2024; Yang S. C. et al., 2025). Compared with international standardized targeted biological agents, domestic research lacks unified efficacy evaluation standards and large-sample randomized controlled clinical trials, leading to low international recognition and difficulty in translational application.

Studies on TCM compound prescriptions focus on elucidating the anti-inflammatory mechanisms of classic formulas. Formulations such as Jingfang Granules, Shufeng San, and Guizhi Fuling Wan can downregulate the levels of inflammatory factors including IL-6, TNF-α, and IL-8 by regulating signaling pathways such as NF-κB, MAPK, and STAT1, thereby ameliorating macrophage polarization imbalance (Huong Nguyen et al., 2022; Jeong et al., 2015; Cao et al., 2026). Shufeng San can also improve behavioral abnormalities in AD mice under stress and reduce central inflammatory factor levels, achieving dual anti-inflammatory effects both systemically and locally (Huong Nguyen et al., 2022). In terms of barrier repair and physical intervention strategies, novel ceramide derivatives, mineral-balanced seawater, and other agents can assist in alleviating AD symptoms by repairing the skin barrier structure and inhibiting macrophage activation, providing new directions for the development of topical preparations (Han and Hyun, 2023; Lee et al., 2018; Kang et al., 2008; Kang et al., 2007).

Domestic research places greater emphasis on translational applications of integrated traditional Chinese and Western medicine. Most natural products have been validated in cellular and animal studies, and preliminary development of topical preparations has been performed for some components. However, research as a whole remains at the preclinical stage. Current studies are limited by several issues, including unclear active ingredients, ambiguous molecular targets, and the lack of standardized quality control systems. Large-sample randomized controlled clinical trials are relatively scarce, which restricts the clinical translation and international recognition of relevant achievements.

## 6 Challenges and future perspectives

### 6.1 Core challenges in clinical translation

Phenotypic heterogeneity of AD represents the primary obstacle to clinical translation. Significant differences exist in interleukin expression profiles and macrophage polarization status in skin lesions among different patients, so the efficacy of single-targeted interventions cannot cover all disease subtypes, and a precise classification and diagnosis system based on biomarkers has not yet been established (Koppes et al., 2016). Insufficient efficiency of local skin targeted delivery constitutes a key technical bottleneck. Most therapeutic agents struggle to penetrate the stratum corneum and act precisely on dermal macrophages. Systemic administration is prone to adverse reactions, while the permeability and stability of topical preparations can hardly meet clinical needs (Lee et al., 2018; Yang S. C. et al., 2025).

Current interventions struggle to achieve long-term efficacy and control disease recurrence. Most regimens only alleviate acute inflammatory symptoms without reversing the pathological memory of macrophage polarization. Inflammation tends to relapse after drug withdrawal, making it difficult to break the pathological cycle at its root (Jang et al., 2023; Kim et al., 2023). Research on natural products and TCM has obvious international research gaps: it lags far behind international biological agent research in systematic mechanism elucidation, component standardization and quality control, and there is a lack of large-sample, multi-center clinical trials conforming to international standards (Huang Y. et al., 2024; Gu et al., 2022; Cao et al., 2026). These problems lead to the low international recognition of TCM research results and restrict its global clinical translation and application.

### 6.2 Future research directions

Individualized stratified therapy will be the core developmental trend in the future. By classifying AD into subtypes based on interleukin expression profiles and macrophage polarization phenotypes, precise intervention strategies targeting specific interleukin-macrophage axes can be developed to improve treatment response rates and achieve personalized therapy (Koppes et al., 2016). The development of highly efficient skin-targeted delivery systems is critical. Novel delivery technologies such as nanocarriers, microneedles and liposomes can be employed to achieve precise delivery of therapeutic agents to dermal macrophages, elevating local drug concentrations while reducing systemic adverse reactions (Lee et al., 2018; Yang S. C. et al., 2025).

#### 6.2.1 Multi-target combination therapy has emerged as a new research trend

Simultaneously targeting pro-inflammatory interleukins and enhancing anti-inflammatory interleukins can achieve the synergistic effects of anti-inflammation, barrier repair and antipruritus, thereby breaking the pathological cycle and reducing the risk of disease recurrence (Jang et al., 2023; Kim et al., 2023). The standardization and mechanistic elucidation of natural products require in-depth and continuous exploration. It is essential to identify the active ingredients, therapeutic targets and signaling pathways of TCM, establish unified quality control standards, and conduct multicenter clinical trials to promote the international application of TCM (Huang Y. et al., 2024; Gu et al., 2022; Cao et al., 2026). Early disease intervention and prevention research deserve significant attention. For high-risk populations of AD, the interleukin-macrophage polarization axis can be regulated to prevent the occurrence and development of the disease, achieving early screening, early intervention and early treatment, thereby reducing the disease incidence rate from the source (Dickel et al., 2010; Hovnanian, 2013).

## 7 Conclusion

The core pathological driver of AD is the dysregulation of the interleukin network, which regulates abnormal macrophage polarization through specific signaling pathways, thereby forming a vicious cycle of inflammatory amplification, skin barrier damage and refractory pruritus that reinforce each other. Regarding the core mechanisms, IL-4 and IL-13 induce macrophage polarization toward a pathologically alternatively activated phenotype by activating the STAT6 pathway. IL-1β, IL-6 (via the NF-κB pathway) and IL-17A (via the STAT1 pathway) drive macrophage polarization toward a pro-inflammatory phenotype. Abnormally polarized macrophages continuously secrete inflammatory mediators such as IL-1β and TNF-α, which directly suppress the expression of key skin barrier proteins including filaggrin and loricrin, mediate pruritic signal transduction, and accelerate the pathological process. In contrast, IL-10 reverses macrophage polarization imbalance by blocking the NF-κB pathway, and IL-22 exerts synergistic anti-inflammatory and barrier-repairing effects by regulating the repair function of keratinocytes.

This study systematically elucidates the complete molecular regulatory mechanism of the interleukin-macrophage polarization axis, identifies the key links where different interleukins precisely regulate macrophage phenotypes through specific signaling pathways, and supplements the research gap in the interactive regulation of immune disorders and barrier damage in AD. It provides a novel therapeutic strategy for blocking the pathological cycle from an upstream level in the disease.

International research has achieved clinical breakthroughs by using targeted biological agents to block key pathways such as IL-4Rα and IL-13, while domestic research has formed distinctive advantages by regulating this axis through multi-targeted natural products. The two approaches complement each other and drive innovations in precision therapy.

Current clinical translation still faces challenges such as disease phenotypic heterogeneity and insufficient efficiency of skin-targeted delivery. Future research should focus on the precise regulation of signaling pathways, optimization of targeted delivery systems and standardization of the mechanisms of natural products, so as to accelerate the clinical translation of this regulatory axis and provide safe and efficient novel therapeutic regimens for severe and refractory AD.
